# Supplementary material for: Complexome profiling of the Chlamydomonas psb28 mutant reveals TEF5 as an early PSII assembly factor
Source: Plant Cell. 2025 Mar 17;37(6):koaf055. doi: 10.1093/plcell/koaf055 (PMC12164590; doi:10.1093/plcell/koaf055)
Supplement: koaf055_Supplementary_Data [file koaf055_supplementary_data.zip › psb28_interactive.zip!/plots/intensity/qualityPlots/peptides/_Cre10.g434200.t1.1.html]

### Cre10.g434200.t1.1

Trivial name:   
SeqCoverage [median]: 54.30  
Mass: 18.84 kDa  
  
 

| Sequence | MissedCleavage | Mass [Da] | wtIntensity | muIntensity | Score |
| --- | --- | --- | --- | --- | --- |
| ADIVSLIDTEK | 0 | 1202.64 | 1954.19 | 4022.75 | 96.89 |
| AFSTAQK | 0 | 751.39 | 734.30 | 0.00 | 76.85 |
| AKQSVTMGER | 1 | 1105.56 | 3313.70 | 1828.12 | 80.83 |
| ALTNPLNHPK | 0 | 1103.61 | 5302.79 | 4931.14 | 122.96 |
| DRADIVSLIDTEK | 1 | 1473.77 | 2081.73 | 4065.25 | 76.76 |
| IAELQLAEQQSFK | 0 | 1503.79 | 7921.53 | 12945.53 | 218.62 |
| KQVQQLEQQLNSK | 1 | 1569.85 | 789.29 | 6739.01 | 110.92 |
| LAELKNEAER | 1 | 1171.62 | 13439.95 | 15509.22 | 93.10 |
| QTDLAAAHQR | 0 | 1109.56 | 591.44 | 0.00 | 39.15 |
| SLALAAAK | 0 | 743.45 | 2827.12 | 4520.74 | 84.24 |
|  |  |  | 38956.0 | 54561.8 |  |
